# Anti-cancer treatment within two weeks serves as a risk factor for clinical outcomes among cancer patients with COVID-19

**DOI:** 10.3389/fonc.2023.1193082

**Published:** 2023-08-22

**Authors:** Jia-Xin Huang, Bo Liu, Xiao-Feng Cong, Yan-Jie Guan, Yi-Qun Zhang, Wei Song, Zhi Li, Zi-Ling Liu, Nan-Ya Wang

**Affiliations:** Cancer Center, The First Hospital of Jilin University, Changchun, Jilin, China

**Keywords:** cancer, Covid-19, anti-cancer treatments, intensive care unit admission, clinical outcome

## Abstract

**Background:**

The coronavirus disease 2019 (COVID-19) pandemic has resulted in infections among patients with cancer. Our study aimed to investigate the potential adverse impact of anti-cancer treatments within 2 weeks of COVID-19 infection on clinical outcomes in patients with cancer.

**Methods:**

This retrospective cohort study analyzed 70 cancer patients with COVID-19 infection from the First Hospital of Jilin University in Changchun City, Jilin Province, between March and June 2022. Data on demographic characteristics, vaccination status, COVID-19 clinical classification, symptoms, complications, tumor-related characteristics, laboratory examinations and medical interventions were extracted from electronic medical record. The primary outcome of our study was Intensive Care Unit (ICU) admission. Logistic regression model was performed to investigate the association between anti-cancer treatments within 2 weeks after COVID-19 infection and the risk of ICU admission.

**Results:**

Of the 70 patients enrolled in this study, 37 received anti-cancer treatments within 2 weeks after COVID-19 infection. Patients receiving anti-cancer treatment were more likely to experience non-mild COVID-19, require oxygen therapy, develop acute respiratory distress syndrome (ARDS) and exhibit elevated inflammatory levels. The risk of ICU admission (P<0.001) and 30-day mortality after reverse transcriptase polymerase chain reaction (RT-PCR) negative conversion (P=0.007) was significantly higher in patients receiving anti-cancer treatments. In multivariate Logistic regression analysis, non-mild classification of COVID-19, anti-cancer treatments within 2 weeks and ECOG > 1were all independently associated with ICU admission after adjusting for confounder factors. The risk of ICU admission rose to 43.63 times (95% confidence interval=1.31–1452.94, P=0.035) in patients receiving anti-cancer treatments within 2 weeks.

**Conclusion:**

Anti-cancer treatments within 2 weeks of COVID-19 infection increase the risk of ICU admission and 30-day mortality after RT-PCR negative conversion in patients with cancer. It may be recommended to postpone cancer-related treatments for more than 2 weeks in cancer patients with COVID-19 infection.

## 1 Introduction

The emergence of the coronavirus disease 2019 (COVID-19) caused by severe acute respiratory syndrome coronavirus-2 (SARS-CoV-2) has had a significant impact on global health since its first reported case in Wuhan in December 2019 (1). The Omicron variant, which emerged in 2022, has further complicated the situation. Cancer patients are particularly vulnerable to infection due to their weakened physical condition and impaired immune system resulting from cancer itself or anti-cancer treatments (2). Previous studies have also confirmed that cancer patients infected with COVID-19 have a higher risk of severe COVID-19 illness and adverse outcomes (3). COVID-19 serves as an accelerator for cancer patients, promoting disease progression and causing adverse disease outcomes. The mortality rate related to COVID-19 in cancer patients is reported to be approximately 25% (4). Therefore, it is crucial to develop appropriate medical interventions for this population.

Patients with cancer require personalized anti-cancer treatments to improve their quality of life and prognosis (5). However, those who are infected with COVID19 may experience adverse effects from anti-cancer treatments. A multicenter cohort study from China reported that patients who underwent surgery were more likely to develop serve clinical presentations, require invasive ventilation and enter to Intensive Care Unit (ICU) (6). A meta-analysis involving 16 studies also showed that active chemotherapy was associated with higher mortality in cancer patients with COVID-19 (7). Patients receiving targeted therapy are suggested to pause for treatment and undergo COVID-19 testing if they develop a fever (8). However, whether to postpone and when to restart anti-cancer treatments for cancer patients diagnosed with COVID-19 are still in debate (9). Liang et al. suggested that delaying curative adjuvant chemotherapy should be taken into account within the accepted duration for each disease site (10). Conversely, some other studies argued against delaying chemotherapy, as it may exacerbate the systemic inflammatory response caused by COVID-19 (11).

The present study aimed to explore the potential adverse impact of anti-cancer treatments within 2 weeks of COVID-19 infection on clinical outcomes in patients with cancer. Various outcomes including ICU admission, clinical outcomes and 30-day mortality after reverse transcriptase polymerase chain reaction (RT-PCR) negative conversion were set to comprehensively evaluate. Our findings would contribute valuable insights for developing clinical guidelines for cancer patients infected with COVID-19.

## 2 Methods

### 2.1 Study population and design

This retrospective cohort study was designed to assess the impact of anti-cancer treatments within 2 weeks of COVID-19 infection on patients with cancer. Data were collected from patients admitted to three designated medical centers of the First Hospital of Jilin University in Changchun, China, from March to June 2022. The inclusion criteria were as follows: (1) 18 years of age or older; (2) positive nasopharyngeal or nasal swab nucleic acid tests results; (3) histopathological or cytological diagnosis of malignancy; (4) history of anti-cancer treatment (e.g., surgery, radiotherapy, chemotherapy, immunotherapy, targeted therapy and palliative care) within the past 4 weeks. Exclusion criteria were as follows: (1) radiological or clinical diagnosis of COVID-19 without a positive RT-PCR testing; (2) lack of important variables required for analysis; (3) survivors who have received radical treatment and have not received any anti-tumor treatment for more than 6 months; (4) severe medical conditions that could interfere with the study results; (5) unable or unwilling to provide informed consent. Patients were divided into two groups: those receiving anti-cancer treatments within 2 weeks of COVID-19 infection including surgery, chemotherapy, immunotherapy, targeted therapy and supportive care and those without any anti-cancer treatments. To collect data on clinical outcomes, telephone follow-up surveys and readmission were applied to all patients. The primary outcome of our study was ICU admission. The secondary outcome was 30-day mortality after RT-PCR negative conversion. This study was approved by the Institutional Review Board of the First Hospital of Jilin University and informed consent was achieved from all patients.

### 2.2 Data collection and variables definition

Patients’ baseline information regarding demographic characteristics, vaccination status, clinical classification of COVID-19, clinical presentation, complications, tumor-related characteristics, laboratory examinations, and medical interventions were extracted from electronic medical records. The severity of COVID-19 was classified as mild or non-mild according to the Ninth Trial Version of COVID-19 Diagnosis and Treatment Guidance (2022) of China (12). Common, severe, and critical COVID-19 cases were all classified into the non-mild group. Patients with underlying conditions such as cardiovascular diseases, chronic lung diseases, diabetes, chronic liver disease, and chronic kidney diseases were considered as high-risk populations. Septic shock, acute respiratory distress syndrome (ARDS), acute kidney injury (AKI), rhabdomyolysis, secondary infection, and other complications were recorded. All of the patients had received traditional Chinese medicine, named *Lianhua Qingwen capsule*, as their treatment regimen for COVID-19. The administration of PAXLOVID was determined based on the individual conditions of the patients. Our study included various solid tumors, including head and neck, lung, gastrointestinal, liver, pancreatic, cervical, ovarian, and other cancers. Eastern Cooperative Oncology Group (ECOG) performance status was used to describe patients’ daily living abilities. Patients who underwent oxygen therapy with high-flow nasal cannula (HFNC), noninvasive positive pressure ventilation (NIPPV), and mechanical ventilation were all recorded. Laboratory data from routine medical tests were also required.

### 2.3 Statistical analysis

Statistical analyses were conducted using SPSS Statistics 26. Continuous variables were expressed as median (interquartile range) and compared using the Mann–Whitney U test. Categorical variables were expressed as absolute numbers or percentages and compared using the χ2 test or Fisher’s exact test. Logistic regression analyses were performed to identify independent predictors associated with ICU admission, with odds ratios (ORs) and 95% confidence intervals (CIs). Variables that were significant at P< 0.05 in the univariate analyses were entered into the multivariate regression models. A two-tailed P-value< 0.05 was considered statistically significant.

## 3 Results

### 3.1 Baseline characteristics

**Figure 1:**
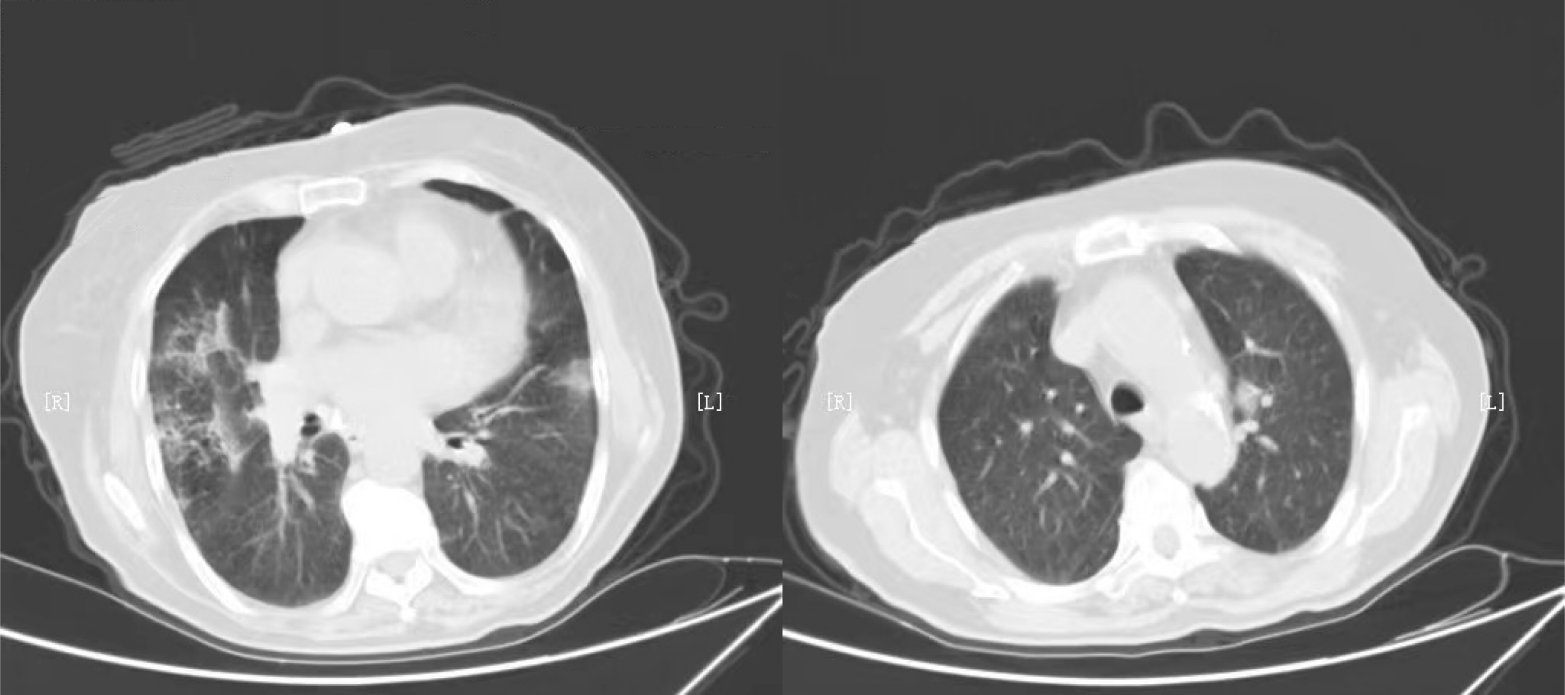
Typical radiological findings of lung in patients with COVID-19.

Seventy cancer patients infected with COVID-19 were totally enrolled in our study, among whom 37 patients received anti-cancer treatments within 2 weeks. The number of patients receiving surgery, radiotherapy, chemotherapy, immunotherapy, targeted therapy and palliative care were 19, 13, 29, 1, 9, and 5, respectively. The median age was 63.0 (IQR=49.5-66.0) years in group receiving treatments and 61.0 (IQR=56.0-70.0) years in group without treatment (P=0.230). Nineteen (51.4%) patients were classified into non-mild COVID-19 in group receiving anti-cancer treatments, significantly higher than that of patients without treatment (P=0.001). Patients were more likely to require special oxygen therapy in group receiving anti-tumor treatments within 2 weeks. The proportion of patients developing ARDS was 16.2% and 0% respectively in two groups, with P value of 0.046. We also observed the value of serum inflammatory markers were higher in patients with anti-cancer treatments within 2 weeks, including NLR (P=0.025), PLR (P=0.048) and IL-6 (P=0.023). There was no significant difference in anti-COVID-19 treatments and tumor site between two groups. More detailed information was showed in **Table 1**. Typical radiological findings of the lung in patients with COVID-19 were displayed in **Figure 1**.

**Table 1:** Patients baseline characteristics.

Variables	Receiving anti-cancer treatments within 2 weeks (n=37)	Receiving no anti-cancer treatments within 2 weeks(n=33)	P value
Age, years	63.0(49.5-66.0)	61.0(56.0-70.0)	0.230
Sex (Male/Female)	19/18(51.4%/48.6%)	14/19(42.4%/57.6%)	0.455
Smoking (Yes/No)	14/23(37.8%/62.2%	11/22(33.3%/66.7%)	0.695
Vaccination (Yes/No)	17/20(45.9%/54.1%)	17/16(51.5%/48.5%)	0.642
High-risk factors (0-1/2-3)	30/7(81.1%/18.9%)	25/8(75.8%/84.2%)	0.588
Hypertension (Yes/NO)	6/31(16.2%/83.8%)	4/29(12.1%/87.9%)	0.883
Diabetes (Yes/No)	2/35(5.4%/94.6%)	5/28(15.2%/84.8%)	0.338
Cerebrovascular disease (Yes/No)	3/34(8.1%/91.9%)	4/29(12.1%/87.9%)	0.873
Coronary heart disease (Yes/No)	4/33(10.8%/89.2%)	3/30(9.1%/90.9%)	1.000
Chronic liver disease (Yes/No)	2/35(5.4%/94.6%)	0/33(0%/100%)	0.494
Clinical presentation (0-1/2-4)	25/12(67.6%/32.4%)	20/13(60.6%/39.4%)	0.544
Fever (Yes/No)	11/26(29.7%/70.3%)	8/25(24.2%/75.8%)	0.606
Cough (Yes/No)	14/23(37.8%/62.2%)	16/17(48.5%/51.5%)	0.369
Dyspnea (Yes/No)	4/33(10.8%/89.2%)	5/28(15.2%/84.8%)	0.854
Fatigue (Yes/No)	3/34(8.1%/91.9%)	4/29(12.1%/87.9%)	0.873
Headache (Yes/No)	5/32(13.5%/86.5%)	1/32(3.0%/97.0%)	0.256
Muscle soreness (Yes/No)	5/32(13.5%/86.5%)	6/27(18.2%/81.8%)	0.592
Sore throat (Yes/No)	9/28(24.3%/75.7%)	9/24(27.3%/72.7%)	0.778
Special oxygen therapy (Yes/No)	20/17(54.1%/45.9%)	6/27(18.2%/81.8%)	0.002*
Nasal catheter oxygen inhalation	20/17(54.1%/45.9%)	6/27(18.2%/81.8%)	0.002*
Non-invasive ventilation	10/27(27.0%/73.0%)	0/33(0%/100%)	0.004*
Mechanical ventilation	2/35(5.4%/94.6%)	0/33(0%/33%)	0.494
Complications (Yes/No)	7/30(18.9%/81.1%)	2/31(6.1%/93.9%)	0.213
Acute respiratory distress syndrome	6/31(16.2%/83.8%)	0/33(0%/100%)	0.046*
Septic shock	1/36(2.7%/97.3%)	0/33(0%/100%)	1.000
Acute kidney injury	0/37(0%/100%)	1/32(3.0%/97.0%)	0.471
Rhabdomyolysis	0/37(0%/100%)	1/32(3.0%/97.0%)	0.471
Classification of COVID-19 (mild/non-mild)	18/19(48.6%/51.4%)	28/5(84.8%/15.2%)	0.001*
Critical COVID-19(Yes/No)	5/32(13.5%/86.5%)	0/33(0%/100%)	0.084
Anti COVID-19 treatments			
Traditional Chinese medicine	30(81.1%)	20(60.6%)	0.104
Traditional Chinese medicine plus PAXLOVID	7(18.9%)	13(39.4%)	
Tumor site (Lung/Others)	16/21(43.2%/56.8%)	14/19(42.4%/57.6%)	1.000
ECOG grade(≤1/>1)	12/25(32.4%/67.6%)	18/15(54.5%/45.5%)	0.062
Gene ORF(Positive/Negative)	19/18(51.4%/13.5%)	17/16(51.5%/48.5%)	0.989
Gene N(Positive/Negative)	22/15(59.5%/40.5%)	18/15(54.5%/45.5%)	0.678
IgMnCOV.IgG(Positive/Negative)	0/37(0%/100%)	2/31(6.1%/93.9%)	0.219
IgGnCOV.IgG(Positive/Negative)	12/25(32.4%/67.6%)	7/26(21.2%/78.8%)	0.292
White blood cell, x $10^{9}$ /L	5.1(3.2-6.0)	4.3(3.0-5.5)	0.212
NLR	3.9(1.6-8.6)	2.5(1.2-3.4)	0.025*
PLR	211.7(151.6-356.5)	158.6(102.8-225.1)	0.048*
Hemoglobin, g/L	114.0(98.5-130.0)	110.0(98.0-130.5)	0.962
Aspartate transaminase, U/L	28.0(20.5-59.0)	21.0(18.0-33.0)	0.025*
Alanine transaminase, U/L	18.0(11.5-34.5)	15.0(10.0-25.5)	0.352
Albumin, g/L	33.6(29.8-38.6)	36.7(31.9-38.6)	0.387
Creatinine, μmol/L	62.0(52.5-82.0)	66.0(52.0-79.5)	0.920
Urea, mmol/L	5.0(3.7-7.3)	5.0(3.8-6.0)	0.592
C-reactive protein, mg/L	11.1(0.0-44.8)	5.7(2.4-15.2)	0.352
IL-6, pg/mL	17.9(10.2-66.0)	11.0(5.4-24.6)	0.023*

RT-PCR, reverse transcriptase polymerase chain reaction; EOCG, Eastern Cooperative Oncology Group performance status; NLR, neutrophil to lymphocyte ratio; PLR, platelet to lymphocyte ratio; IL, interleukin. * P value < 0.05 was considered statistically significant.

### 3.2 Outcomes of the present study

The present study set two outcomes, including ICU admission and 30-day mortality after RT-PCR testing negative conversion, to comprehensively evaluate the impact of anti-cancer treatment within two weeks of COVID-19 (**Table 2**). In group with anti-cancer treatments, 19 (51.4%) patients were admitted to the ICU, which was significantly higher than that of patients without anti-cancer treatments (P<0.001). The 30-day mortality rate after RT-PCR negative conversion was 24.3% and 0% in patients with and without anti-cancer treatments, respectively (P=0.007).

**Table 2:** Outcomes between patients with and without anti-cancer treatments within 2 weeks.

Variables	Receiving anti-cancer treatments within 2 weeks (n=37)	Receiving no anti-cancer treatments within 2 weeks(n=33)	P value
ICU admission			<0.001*
Yes	19(51.4%)	1(3.0%)	
No	18(48.6%)	32(97.0%)	
30-day mortality after RT-PCR negative conversion			0.007*
Yes	9(24.3%)	0(0%)	
No	28(75.7%)	33(100%)	

RT-PCR, reverse transcriptase polymerase chain reaction; ICU, intensive care unit. * P value < 0.05 was considered statistically significant.

### 3.3 Univariate and multivariate logistic regression analysis of ICU admission

We performed logistic regression analysis to explore the association between anti-cancer treatments within 2 weeks of COVID-19 infection with ICU admission in patients with cancer (**Table 3**). Patients using special oxygen therapy, having complications, developing non-mild classification of COVID-19, receiving anticancer treatments within 2 weeks or with ECOG grade above 1 were more likely to enter ICU. The non-mild classification of COVID-19, anti-cancer treatments within 2 weeks and ECOG above 1 were confirmed to be independent risk factors associated with ICU admission after adjusting for confounding factors. The risk of entering ICU rose to 43.63 times (95%CI=1.31–1452.94, P=0.035) in patients receiving anti-cancer treatments within 2 weeks.

**Table 3:** Univariate and multivariate Logistic regression analyses of ICU admission.

Variables	Univariate		Multivariate	
	Hazard ratio(95%CI)	P value	Hazard ratio(95%CI)	P value
Age, years	1.02(0.97-1.07)	0.437		
Sex (Male/Female)	1.56(0.55-4.41)	0.406		
Smoking (Yes/No)	0.70(0.23-2.13)	0.529		
Vaccination (Yes/No)	0.62(0.22-1.76)	0.366		
High-risk factors (0-1/2-3)	0.75(0.22-2.56)	0.646		
Clinical presentation (0-1/2-4)	2.00(0.63-6.38)	0.241		
Special oxygen therapy (Yes/No)	25.82(6.22-107.20)	<0.001*	15.22(0.67-344.57)	0.087
Complications (Yes/No)	12.92(2.39-69.81)	0.003*	44.29(0.29-6663.25)	0.138
Time to RT-PCR negative, days	0.98(0.90-1.06)	0.555		
Classification of COVID-19 (mild/non-mild)	0.03(0.01-0.12)	<0.001*	0.02(0.00-0.39)	0.009*
Anti-cancer treatments within 2 weeks (Yes/No)	33.78(4.17-273.68)	0.001*	43.63(1.31-1452.94)	0.035*
Anti COVID-19 treatments (Traditional Chinese medicine plus PAXLOVID/Traditional Chinese medicine)	0.20(0.03-0.79)	0.043*	0.37(0.01-14.45)	0.595
Tumor site (Lung/Others)	1.50(0.53-4.31)	0.446		
ECOG grade(≤1/>1)	0.04(0.01-0.31)	0.002*	0.002(0.00-0.91)	0.047*
Gene ORF(Positive/Negative)	0.92(0.33-2.61)	0.880		
Gene N(Positive/Negative)	0.89(0.31-2.52)	0.819		
IgMnCOV.IgG (Positive/Negative)	NE	0.999		
IgGnCOV.IgG (Positive/Negative)	1.71(0.55-5.25)	0.352		

Those variables found significant at P< 0.05 in the univariate analyses were entered into the multivariate Cox regression analyses.

RT-PCR, reverse transcriptase polymerase chain reaction; EOCG, Eastern Cooperative Oncology Group performance status. * P value < 0.05 was considered statistically significant.

## 4 Discussion

This study, for the first time, investigated the impact of anti-cancer treatments within two weeks of COVID-19 infection on clinical outcomes among patients with cancer. We retrospectively analyzed 70 cancer patients with COVID-19 infection, with 37 patients receiving anti-cancer treatments including surgery, chemotherapy, immunotherapy, targeted therapy and palliative care within two weeks after COVID-19 infection confirmed by RT-PCR testing. The baseline characteristics were virtually balanced in both groups, except the risk of developing non-mild COVID-19, requiring special oxygen therapy, suffering acute respiratory distress syndrome (ARDS) and being in increased inflammatory status. No statistically significant difference in anti-COVID-19 treatments and tumor site was found between two groups. We set two outcomes including ICU admission and 30-day mortality after RT-PCR negative conversion, to comprehensively assess the impact of anti-cancer treatments within two weeks on cancer patients with COVID-19 infection. The results of our study showed that anti-cancer treatments within two weeks after COVID-19 infection were associated with higher risk of ICU admission and 30-day mortality after RT-PCR negative conversion. Logistic regression analysis also found it served as an independent unfavorable factor for ICU admission adjusting for confounders.

Previous researches on whether anti-cancer treatments lead to a worse clinical outcome in COVID-19 patients with cancer have been inconsistent. We speculated it may be because the timing of anti-cancer treatments is not taken into consideration, which is an important factor for physicians to consider when providing medical intervention in clinical practice. Lee et al. reported that the mortality of patients who received immunotherapy, hormonal therapy, targeted therapy, and radiotherapy within the past 4 weeks before COVID-19 infection had no significant difference from patients without anti-cancer treatments (13). Similarly, a meta-analysis consisting of 29 studies showed that all types of anti-tumor therapy within 3 months before diagnosis of COVID-19 had no significant effect on mortality and ICU admission rate in solid tumor patients (5). These results only indicate that anti-cancer treatments before COVID-19 infection have no influence on mortality in cancer patients. However, our study indicated that the risk of ICU admission and 30-day mortality after RT-PCR negative conversion would significantly increase if patients receive anti-cancer treatments within 2 weeks after the diagnosis of COVID-19. Consistent with our study, Tanabe et al. reported that before the discharge criteria were met and all symptoms had disappeared, chemotherapy should not be restarted in post-COVID-19 patients who had experienced mild illness (14). Experts from Australia and New Zealand advised that minor surgery should be postponed for at least 4 weeks and major surgery for 8-12 weeks after laboratory confirmation of SARS-CoV-2 (15). The China Anti-Cancer Association suggested that the optimal timing of continuing anti-tumor treatments is when symptoms have completely alleviated, and two consecutive RT-PCR tests are both negative (with an interval time exceeding 24 hours) (16). Large-scale randomized clinical research is required to explore the optimal timing for resuming cancer treatments in COVID-19 patients. Results from our study showed that anti-cancer interventions within 14 days after COVID-19 infection are too early for patients, with no benefit but worse clinical outcomes.

Our study included various anti-cancer treatments, including surgery, chemotherapy, immunotherapy, targeted therapy and palliative care, but their effects on outcomes may differ due to their distinct mechanisms. The process of mechanical ventilation, anesthesia and tissue damage involved in surgery may further promote inflammatory response and suppress the immune system caused by SARS-CoV-2 infection (17). Additionally, respiratory complications related to surgery may increase the risk of worse clinical outcomes (18). Chemotherapy drugs can suppress the immune system of our body when disrupting tumor cell proliferation and lead to poor physical conditions (19). Patients receiving active chemotherapy have been confirmed to be susceptible to respiratory infections (20). Moreover, chemotherapy and targeted therapy could exacerbate the cytokine storm caused by SARS-CoV-2, which can lead to acute respiratory distress syndrome (ARDS) (5). This is consistent with what we found in our study, that patients receiving anti-cancer treatments were at an elevated inflammation status and more prone to develop complications such as ARDS. Previous studies have also shown that patients receiving surgery and chemotherapy in the month before COVID-19 infection had increased odds of suffering adverse clinical events (10). On the other hand, some studies have shown that patients receiving radiotherapy and immunotherapy had no significant association with severe events when infected with COVID-19 (4, 6). Vivarelli et al. also hypothesized that immune checkpoint inhibitors (ICIs) could serve as a protective factor for cancer patients by reactivating T-cells to kill tumor cells as well as virus-infected cells (21). However, further studies are required to clarify the detailed interplay between different anti-tumor therapies and SARS-CoV-2.

We also found that the severity of COVID-19 and ECOG were both independently associated with ICU admission in patients with cancer. The risk of entering ICU was higher in those patients with non-mild COVID-19 and EOCG above 1. These findings are consistent with previous study which reported that age, comorbidities, and concurred medication use all influenced survival of patients with cancer (22). Poor performance status is related to adverse outcomes such as lower tolerance to anti-cancer treatment, poor quality of life, and decreased survival (23). Of note, a patient’s functional status can quickly change when infected with COVID-19. Therefore, it is of vital importance to monitor their performance status closely and formulate personalized treatments.

Several limitations of the present study we have admitted. First, as a retrospective study, our study may be influenced by potential selection bias. In group with anti-cancer treatments within 2 weeks, the proportion of patients developing non-mild classification of COVID-19 was significantly higher than group without treatments. Moreover, asymptomatic COVID-19 patients with cancer did not be taken into consideration because they would not take RT-PCR testing without clinical presentations. Although we performed multivariate Logistic regression adjusting for confounder factors, there may be other potential risk factors we ignored in the present study. Second, patients with surgery, radiotherapy, chemotherapy, immunotherapy, targeted therapy and palliative care were all classified into group with anti-cancer treatments. The interpretation should be further validated in specific anti-cancer treatment considering different treatment may have a different impact on prognosis. Third, we only showed anti-cancer treatments within 2 weeks after SARS-CoV-2 infection were detrimental to clinical outcomes, but the optimal interrupted-interval and restart timing are still unknown.

In conclusion, our present study showed anti-cancer treatments within 2 weeks of COVID-19 diagnosis increase the risk of ICU admission and 30-day mortality after RT-PCR negative conversion for patients with cancer. We would recommend delaying cancer-related treatments for more than 2 weeks after COVID-19 diagnosis in clinical practice.

## Data availability statement

The original contributions presented in the study are included in the article/supplementary material. Further inquiries can be directed to the corresponding author.

## Ethics statement

The studies involving humans were approved by the Institutional Review Board of the First Hospital of Jilin University. The studies were conducted in accordance with the local legislation and institutional requirements. The participants provided their written informed consent to participate in this study.

## Author contributions

All authors read and approved the final manuscript. N-YW and Z-LL conceived and designed the study. J-XH, BL X-FC, and Y-JG assisted with the development of the methods. J-XH, BL, and Y-QZ did the data analysis. J-XH and WS drafted the initial manuscript. ZL, WS, Y-QZ, Y-JG, Z-LL, and N-YW gave many valuable comments on the draft and polished it. All authors assisted with the interpretation of the findings, commented on drafts of the manuscript, and approved the final version. All authors contributed to the article.
